# Lateralized brain activities in subcortical vascular mild cognitive impairment with differential Chinese medicine patterns: A resting-state functional magnetic resonance imaging study

**DOI:** 10.3389/fnins.2022.943929

**Published:** 2022-08-22

**Authors:** Jianjun Wang, Fanxin Kong, Haotao Zheng, Dongbin Cai, Lijin Liu, Jie Lian, Hanqing Lyu, Songjun Lin, Jianxiang Chen, Xiude Qin

**Affiliations:** ^1^Department of Neurology and Psychology, Shenzhen Traditional Chinese Medicine Hospital, Shenzhen, China; ^2^The Fourth Clinical Medical College, Guangzhou University of Chinese Medicine, Shenzhen, China; ^3^Harvard Medical School, Global Clinical Scholars Research Training (GCSRT), Boston, MA, United States; ^4^Department of Radiology, Shenzhen Traditional Chinese Medicine Hospital, Shenzhen, China

**Keywords:** subcortical vascular mild cognitive impairment, fALFF, fMRI, Chinese medicine, syndrome differentiation, deficiency pattern, excess pattern, lateralization

## Abstract

**Background:**

Subcortical vascular mild cognitive impairment (svMCI) is one of the most treatable cognitive impairments, but could be hampered by the high clinical heterogeneities. Further classification by Chinese Medicine (CM) patterns has been proved to stratify its clinical heterogeneities. It remains largely unknown of the spontaneous brain activities regarding deficiency patterns (DPs) and excess patterns (EPs) of svMCI patients based on fMRI data.

**Objective:**

We aim to provide neuroimaging evidence of altered resting-state brain activities associated with DPs and EPs in svMCI patients.

**Methods:**

Thirty-seven svMCI patients (PAs) and 23 healthy controls (CNs) were consecutively enrolled. All patients were categorized into either the EP group (*n* = 16) and the DP group (*n* = 21) based on a quantitative CM scale. The fractional amplitude of low-frequency fluctuation (fALFF) value was used to make comparisons between different subgroups.

**Results:**

The DP group showed significant differences of fALFF values in the right middle frontal gyrus and the right cerebellum, while the EP group showed significant differences in the left orbitofrontal gyrus and the left cerebellum, when compared with the CN group. When compared with the EP group, the DP group had markedly increased fALFF values in the left superior temporal gyrus, right middle temporal gyrus and brainstem. The decreased fALFF values was shown in the right anterior cingulate and paracingulate gyri. Among the extensive areas of frontotemporal lobe, the Montreal Cognitive Assessment (MoCA) scores were significantly correlated with the reduced fALFF value of the right middle frontal gyrus and the left orbitofrontal gyrus.

**Conclusion:**

Our results indicated that the DPs and EPs presented the lateralization pattern in the bilateral frontal gyrus, which will probably benefit the future investigation of the pathogenesis of svMCI patients.

## Introduction

Subcortical vascular mild cognitive impairment (svMCI) refers to mild cognitive disorder with underlying subcortical lacunar stroke or white matter hyperintensities (WMHs) (T O’Brien et al., 2003). It is one of the most treatable dementia (Rockwood et al., 2000), and has been proposed as a relatively homogeneous subtype of all causes of vascular dementia. However, clinical presentations often differ greatly because of variations in differential subsets of conventional vascular neuroimaging structure (e.g., lacunar and CMBs with differential location, size, and volume, etc.). The resting-state functional magnetic resonance imaging (rs-fMRI) is an emerging and non-invasive technique to detect the brain intrinsic functional architecture and meanwhile overcome the unbalanced distribution of conventional vascular lesions (Vinciguerra et al., 2020; Wang R. et al., 2020). Inspired by the milestone fMRI study to establish subtypes for heterogeneous disease (Drysdale et al., 2017), there is much interest in using rs-fMRI to explore the association of phenotypes and neurobiological subtypes in svMCI.

Interestingly, it is the essence of Chinese medicine (CM) and prerequisite of CM treatment to classify the same biological disease into different etiopathological patterns/subtypes (Tang et al., 2008; Lu et al., 2012). A total of 196 CM patterns have been included in the latest International Classification of Diseases 11th version (ICD-11) coding system (WHO, 2019). Among them, deficiency pattern (DP) and excess pattern (EP), consisting of two contrary and complementary clinical manifestations, have been utilized as two basic patterns/subtypes to ensure reasonable treatment of diseases (Tang et al., 2008). This strategy is especially effective for complicated diseases and has been used in China, Japan, Korea, and elsewhere worldwide (Lam et al., 2019). In a multicenter cohort study of vascular cognitive impairment, DPs exhibited significantly lower naming factor scores relative to EPs (Zheng et al., 2022a), sharpening specific clinical characteristics and treatment targets. The underlying neural mechanisms regarding DPs and EPs in svMCI patients remain to be further elucidated.

It is increasingly accepted that fMRI could help to understand the neural basis behind different CM patterns of disease. Taking major depression for example, the EP subtype is often comorbid with nervousness and irritability, whereas the DP subtype is characterized by excessive pensiveness, suspicion, and timorousness. The functional connectivity of the insular (Liu et al., 2019) and posterior cingulate cortex (Zhang et al., 2015) was found to explain the neural basis of the above clinical alterations, and indicated the sad-face processing variation between DPs and EPs of major depression (Wang et al., 2019). There were also fMRI studies demonstrating the altered resting-state brain activities with different CM patterns in depression with anxiety (Xu et al., 2018; Du et al., 2020) and psychogenic erectile dysfunction (Liu et al., 2015). These studies indicate the methodological possibilities for researching altered cerebral activities based on DPs and EPs in svMCI patients. Further, as an advancing approach to detect spontaneous brain activity with much higher sensitivity and specificity (Zou et al., 2008), the fractional amplitude of low-frequency fluctuations (fALFF) value provides a promising avenue for exploring cerebral alterations that are associated with symptomatic variations between DPs and EPs in svMCI patients.

Taking together, the current study aimed to provide physiological evidence, using functional magnetic resonance imaging (fMRI), to identify altered resting-state brain activity associated with DP and EP patterns in svMCI patients. We hypothesize that fALFF values in the DP and EP subgroups of svMCI patients (PAs) were significantly altered compared with healthy controls (CNs). To test this hypothesis, we examined fALFF value differences between (1) PAs and CNs, (2) DPs and CNs, EPs vs. CNs, and DPs vs. EPs.

## Material and methods

### Participants

We consecutively recruited a total of 60 right-handed participants (Han Chinese) from February 2017 to January 2019. They consisted of 37 svMCI patients and 23 demographically matched healthy controls. All svMCI patients were enrolled from a previous randomized control study (Zheng et al., 2022b), whereas healthy participants were enrolled from community residents by advertisements. Complete recruitment details were described in our prior work (Lyu et al., 2019; Wang J. et al., 2020; Xu J. et al., 2020; Xu Z. et al., 2020). Briefly, the diagnosis criteria of svMCI were performed according to Petersen’s criteria (Petersen, 2004). T2 FLAIR images showed WMHs with a Fazekas rating scale score ≥ 2, or multiple (> 3) supratentorial subcortical small infarcts (< 20 mm), or one/more subcortical small infarcts in the caudate nucleus, Globus pallidus, or thalamus (Jia et al., 2016). We excluded patients presenting secondary causes of cognitive deficits according to previously described criteria (Román et al., 2002; Moorhouse and Rockwood, 2008; Jia et al., 2016). Healthy controls had no history of any neurological or psychiatric disorders, no cognitive complaints, and no abnormalities on their conventional brain MRI images. All subjects provided written informed consent to participate in the study. All aspects of this study were approved by the Institutional Review Board of Shenzhen Traditional CM Hospital.

### Clinical measures

All subjects underwent a clinical evaluation that assessed their demographic characteristics (age, sex, and level of education), various vascular risk factors (smoking, alcohol, hypertension, diabetes mellitus, and body mass index), as well as brain MRI scanning. All participants underwent a standardized neurological examination and assessment of the Beijing version of the Montreal Cognitive Assessment (MoCA). The scale for the differentiation of syndromes of vascular dementia (SDSVD) = was developed in 2002 (Tian et al., 2002) to power the standardized evaluation of CM syndrome in trials of vascular cognitive impairment (Shi et al., 2014). It was included in the present study to quantitatively assess all patients and divided them into four subtypes, namely kidney essence deficiency (KED), qi and blood deficiency (QBD), phlegm obstruction of orifices (POO), and stasis blocking channels (SBC). KED and QBD are representative of DPs, and POO and SBC are representative of EPs. Each subtype is assigned a score from 0 to 30, with a sum score of at least 7 indicating confirmation of the subtype. If the patients meet more than 2 subtypes, the subtype with the highest score is labeled as the dominant CM pattern, by which the DPs or EPs will be determined (Tian et al., 2002).

### Functional magnetic resonance imaging data acquisition

MRI images were acquired using a GE Discovery MR750 3.0T MRI scanner (General Electric Medical Systems, Milwaukee, WI, United States) at the Shenzhen Hospital of Traditional CM. For all subjects, high-resolution structural images were acquired with a three-dimensional MRI sequence using an axial fast spoiled gradient recalled sequence with the following parameters: repetition time (TR) = 8.62 ms, echo time (TE) = 3.224 ms, flip angle (FA) = 12°, data matrix = 512 × 512, field of view (FOV) = 256 × 256 mm^2^, and continuous sagittal slices = 152 with 1 mm thickness. Functional images were acquired with an echo-planar imaging (EPI) sequence with the following parameters: TR = 2,000 ms, TE = 35 ms, FA = 90°, data matrix = 64 × 64, resolution = 3.75 × 3.75 mm^2^, slices thickness = 4 mm with no inter-slice gap, and volumes = 240 with 38 axial slices. All subjects were required to lie flat, close their eyes, and relax while remaining awake during the scanning process. After the data scanning, all subjects verified that they remained awake during the scan, and the neuroimaging quality was checked.

### Functional magnetic resonance imaging data processing

Data preprocessing was performed using statistical parametric mapping (SPM8)^1^ (Friston et al., 1994) and DPABI^2^ (Yan et al., 2016), following standardized principles and quality control procedures (Polli et al., 2016; Takeuchi et al., 2017; Chen et al., 2018). First, the first 10 volumes in the time series were discarded to avoid non-equilibrium effects in the MR signal. Then the functional images were slice-timing corrected, which was performed by interpolating the voxel time using slice interpolation. Next, all functional images were spatially realigned and co-registered to their corresponding anatomical images. Then, the resulting images were spatially normalized to Montreal Neurological Institute (MNI) space, resampled to 3 mm × 3 mm × 3 mm voxels and further spatially smoothed using a Gaussian kernel with 6 mm full-width at half maximum (FWHM) (Tahmasebi et al., 2009). Finally, potential sources of 24 head motion parameters, global signals (GSs), white matter (WM) signals and cerebrospinal fluid (CSF) signals were regressed out to remove their effects (Murphy et al., 2009). Particularly, given a possible confounding effect of micromovements (Dijk et al., 2012), the framewise displacement (FD) values, which reflected the temporal derivative of the movement parameters (Power et al., 2012; Yang et al., 2014), were calculated for each subject. One subject with svMCI and two healthy subjects who had a mean FD = 0.5 mm or translation > 2 mm or rotation > 2 degrees were excluded. In total, data from the remaining 37 patients with svMCI and 23 healthy controls were used for further analysis.

### Fractional amplitude of low-frequency fluctuation analysis

After preprocessing, the linear trend was removed and fALFF analysis was carried out using DPABI software (Yan et al., 2016). The analysis procedure for fALFF was carried out according to the method of previous studies (Zou et al., 2008; Liu et al., 2017). fALFF values were computed in a voxel-wise manner using volumetric fMRI data. The BOLD time series of all brain voxels were converted to the frequency domain *via* the fast Fourier transform (FFT; MATLAB), and normalized power spectrums were subsequently obtained. The fALFF index was computed in a voxel-wise manner as the sum of power in the 0.01–0.1 Hz frequency band divided by the sum of power of the entire frequency range (0.01–0.25 Hz). Finally, the subject-level voxel-wise fALFF maps were standardized into subject-level Z-score maps by subtracting the mean voxel-wise fALFF obtained for the whole brain and dividing by the standard deviation.

### Statistics analysis

Participants’ baseline characteristics, including demographic information (i.e., age, sex, and body mass index) and clinical scores (i.e., SDSVD and MoCA) were compared between groups. Two-tailed two-sample *t*-tests were performed to examine the significant group-level differences between the PA group and CN group, DP/EP group and CN group, as well as differences between the DP group and EP group. The statistical significance level was set at *p* < 0.05. All statistical tests were performed in IBM SPSS Statistics 21.0 software.

To investigate the effect of differences in fALFF values at the group level, a two-tailed two-sample *t*-test was performed on the individual fALFF maps between different groups (PAs vs. CNs, DPs vs. CNs, EPs vs. CNs and DPs vs. EPs). In particular, age, sex and education level were considered variables of no interest and were regressed out to remove their effects. The threshold of significance was set at a *p* = 0.05 combined with correction for multiple comparisons using the AlphaSim method (a minimum cluster threshold of 178 voxels of 3-mm cubic in MNI space).

Moreover, to identify the relationship of the fALFF values in regions with significant group-level differences and clinical characteristics, the mean fALFF values were calculated first. Then, Pearson’s correlation analysis was performed between the mean fALFF values and the cognitive performance of the patients (MoCA).

## Results

### Demographic and clinical characteristics of subjects

A total of 37 svMCI patients and 23 healthy controls were included for analysis in the present study. Thirty-seven svMCI patients in the PA group were further divided into the EP group (*n* = 16) and DP group (*n* = 21). Demographic and clinical data for all subjects are shown in Table 1. MoCA scores were significantly lower in the PA group, DP group and EP group than that in the CN group. The ratio of hypertension in both the DP and EP groups, as well as the education level and age in the DP group, were significantly different from those in the CN group.

**TABLE 1:** Demographic and clinical characteristics in the PA group, DP group, EP group and CN group.

Groups	PA (*N* = 37)	DP (*N* = 21)	EP (*N* = 16)	CN (*N* = 23)	*p*-value			
					PA vs. CN	DP vs. CN	EP vs. CN	DP vs. EP
Gender (male/female)^a^	19/18	9/12	10/6	9/14	0.36	0.80	0.15	0.24
Age (years, mean ± SD)^b^	63.86 ± 6.91	65.67 ± 6.38	61.50 ± 7.05	61.91 ± 4.86	0.24	0.03*	0.83	0.07
Education (years, mean ± *SD*)^b^	8.27 ± 3.73	7.33 ± 3.09	9.50 ± 4.23	9.83 ± 3.55	0.12	0.02*	0.80	0.08
BMI (mean ± *SD*)^b^	23.63 ± 2.73	23.47 ± 2.61	23.84 ± 2.96	23.17 ± 2.36	0.51	0.69	0.44	0.69
Smoking (yes/no)^a^	15/22	9/12	6/10	4/19	0.06	0.06	0.26^△^	0.74
Alcohol (yes/no)^a^	9/28	5/16	4/12	4/19	0.75^△^	0.72^△^	0.69^△^	0.93^△^
Hypertension (yes/no)^a^	26/11	14/7	12/4	5/18	<0.001*	0.003*	0.001*	0.58^△^
Diabetes mellitus (yes/no)^a^	13/24	5/16	8/8	4/19	0.14	0.72^△^	0.04^△^	0.10
Hyperlipidemia (yes/no)^a^	10/27	5/16	5/11	3/20	0.33^△^	0.45^△^	0.24^△^	0.61
MoCA (mean ± *SD*)^b^	19.49 ± 2.06	18.95 ± 1.91	20.19 ± 2.10	28.83 ± 1.11	<0.001*	<0.001*	< 0.001*	0.07

^a^Binary variables were analyzed using the chi-square test.

^b^Quantitative parameters were analyzed using a two-sample t-test.

*Represents a significant difference between the two groups.

^△^ Fisher’s exact test.

PA, patient; DP, deficiency pattern; EP, excess pattern; CN, control; BMI, body mass index; MoCA, Montreal cognitive assessment.

### Group comparisons

**FIGURE 1:**
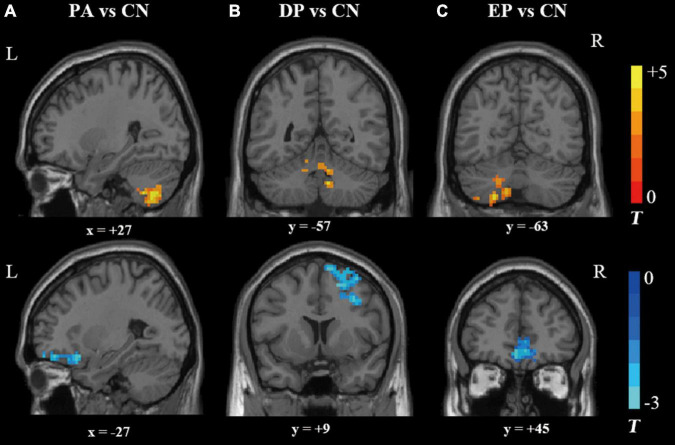
Brain regions that showed significant alterations in fALFF values. **(A)** the PA group vs. the CN group, **(B)** the DP group vs. the CN group; and **(C)** the EP group vs. the CN group. fALFF, fractional amplitude of low-frequency fluctuation; PA, patient; DP, deficiency pattern; EP, excess pattern; CN, control; R, right; L, left.

The results obtained from the two-sample *t*-test clearly demonstrated that there were significant differences between different groups when comparing each patient group to the CN group. The PA group exhibited significantly reduced fALFF values in the left superior and medial frontal gyrus, and significantly increased fALFF values in the right cerebellum (Supplementary Table 1 and Figure 1A). However, when the PA group was divided into the DP and EP groups, the DP group exhibited significantly reduced fALFF values in the right middle frontal gyrus, and significantly increased fALFF values in the right cerebellum (Supplementary Table 1 and Figure 1B). In contrast, the EP group exhibited significantly reduced fALFF values in the left orbitofrontal gyrus, and increased fALFF values in the left cerebellum (Supplementary Table 1 and Figure 1C). Further, when the DP group was compared to the EP group, we found significantly increased fALFF values in the left superior temporal gyrus, right middle temporal gyrus, and brainstem, and significantly decreased fALFF values in the right anterior cingulate and paracingulate gyri (Supplementary Table 1 and Supplementary Figure 1).

### Correlation analysis

**FIGURE 2:**
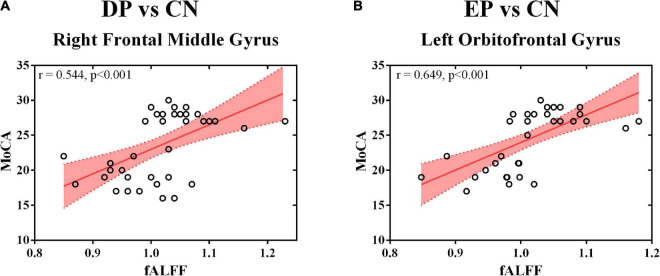
Correlation analysis. **(A)** The fALFF value of the right frontal middle gyrus and the MoCA score; **(B)** The fALFF value of the left orbitofrontal gyrus and the MoCA score. fALFF, fractional amplitude of low-frequency fluctuation; DP, deficiency pattern; EP, excess pattern; CN, control; MoCA, the Montreal Cognitive Assessment.

The MoCA scores were positively correlated with the fALFF value of the right frontal middle gyrus (*r* = 0.544, *p* < 0.001) (Figure 2A) and the left orbitofrontal gyrus (*r* = 0.649, *p* < 0.001) (Figure 2B). Non-significant correlations were observed between the MoCA scores and the fALFF value of any interested gyrus in the comparison of DP and EP. The whole correlation analysis results can be found in Supplementary Table 2.

## Discussion

In the present study, we measured fALFF value alterations before and after svMCI patients were categorized into DP and EP subgroups. We found altered resting-state brain activities in cerebellum, brainstem and widespread frontotemporal area extending to the right middle frontal gyrus, right anterior cingulate gyrus, right middle temporal gyrus, left superior temporal gyrus, and right anterior cingulate and paracingulate. The lateralized activation of the right middle frontal gyrus and the left orbitofrontal gyrus were found steady and significantly correlated with the MoCA scores. Our findings provide the initial evidence of lateralized brain activation of two contrary CM patterns of DPs and EPs in svMCI patients. These finding might extend our present understanding and allow better stratification of heterogeneous svMCI patients.

We repeatedly found that svMCI patients showed lower prefrontal fALFF values and higher cerebellar fALFF values both before and after the classification of CM patterns. The frontal lobe is one of the most important brain regions subserving cognitive regulation (MacDonald et al., 2000; Arnsten et al., 2021). Dysregulation of the dorsolateral prefrontal cortex leads to clinical cognitive decline and provides the most commonly used targets for non-invasive brain stimulation (Birba et al., 2017). The increased fALFF values were associated with significant improvement of cognitive function after treatment (Qin et al., 2022). According to the “disconnection hypothesis” (Galluzzi et al., 2008), disruption by WMHs or lacunar infarcts of the prefrontal-frontal circuits implicated in the cortical loops and interhemispheric connectivity may primarily result in cognition decline (Puglisi et al., 2018; Vinciguerra et al., 2019; Cantone et al., 2020). Thus, it was not surprising that we found altered frontal fALFF values in svMCI patients due to vascular lesions leading to disrupted plasticity (Yi et al., 2012). Further, we found the increased activities of cerebellar significantly correlated with cognition performance, which could be supported by the recent paradigm shift of cerebellar neuroscience involved in cognition regulation (Schmahmann et al., 2019; Lin et al., 2022). Our study supported the robust biological findings in svMCI patients even in different CM patterns.

It is interesting that we observed opposite sides of brain activation corresponding to DPs and EPs in svMCI patients. Especially, the fALFF value of bilateral clusters of interest was significantly correlated with the disease severity. It is known that EPs and DPs have been clinically utilized for subtype identification and treatment decision making for thousands of years (Jiang et al., 2012; Lee et al., 2015). Functional MRI-derived brain activity has been introduced to explore this mystery based on the development of cognitive neuroscience (Liu et al., 2015; Xu et al., 2018). Consistent with the lateralized pattern in our study, the main differences in posterior cingulate cortex (PCC) functional connectivity between EPs and DPs occurred in the left PCC in depressive patients (Zhang et al., 2015). Meanwhile, we found that the differences in EPs vs. CNs and DPs vs. CNs were totally different from the findings in DPs vs. EPs. This tendency was replicated in functional connectivity analysis in major depression (Zhang et al., 2015), which arises further interests in exploring neural mechanisms behind different CM patterns. Currently, the cell type-specific signature genes were found to significantly correlate with cerebral cortical differences (Li et al., 2021), which provided a novel approach to interpret our findings based on the gene enrichment of the difference map of EPs and DPs. Notably, whenever compared with CNs or EPs, DPs showed decreased resting-state brain activity in the right middle frontal gyrus and anterior cingulate gyrus. These regions are critical for intrinsic connectivity networks that mediated cognitive processing (Zhao et al., 2019). Especially, the reduced activity of the right middle frontal gyrus contributed to the worse cognitive performance. This was of great practical significance because the classical CM theory holds the view that the DP patients always exhibit a long-lasting course of disease with concomitant cognitive impairment (Lin et al., 2020; Wang B. Q. et al., 2020). Interestingly, Ning et al. (2018) demonstrated that the DP individuals had memory and attention impairments by nature even in healthy subjects, to which the abnormal functional connectivity of the executive control network was attributed. Thus, our study provided neuroimaging response for DPs in svMCI patients and extended our insights into CM patterns with cerebral plasticity.

There are several limitations of this study. First, as participants were recruited from a previous randomized control study (Zheng et al., 2022b) and composed of only partial CM patterns defined by SDSVD, the altered neural activities behind all DPs and EPs should be explained with caution considering the selection bias. Second, the relatively small sample size and potential confounders (e.g., unclear medication consumption) might have compromised the statistical power and the external validity of the analysis. Third, all patients had numerous neuroimaging lesions with small subcortical infarcts and/or randomly distributed WMHs. We cannot exclude the potential effects of these lesions, since it was difficult to assess the randomly distributed location and relatively small volume of all the lesions.

## Conclusion

The “DP and EP” theory has long time been utilized for patient treatment in the field of CM. The present study showed a lateralized neural activation paradigm in the bilateral frontal gyrus that may distinguish DPs from EPs in svMCI patients. The right middle frontal gyrus might serve as a brain response to endogenic cognitive impairments of DPs in svMCI patients. Understanding the neural mechanism underlying differential CM patterns will probably benefit the future investigation of the pathogenesis of svMCI, which might improve treatment approaches.

## Data availability statement

The datasets generated for this study are available on request to the corresponding authors.

## Ethics statement

The studies involving human participants were reviewed and approved by the Ethics Committee of Shenzhen Traditional Chinese Medicine Hospital. The patients/participants provided their written informed consent to participate in this study.

## Author contributions

JW, SL, HZ, LL, HL, FK, JC, and XQ: conceptualization and writing—review and editing. JC, JL, and LL: data curation. HZ, DC, and LL: formal analysis. JW, FK, and XQ: funding acquisition. HZ, LL, DC, JL, HL, and FK: investigation. JC, JW, SL, and XQ: methodology. JC, SL, and XQ: supervision. JW and HL: visualization. JW and JC: writing—original draft. All authors contributed to the article and approved the submitted version.

## Footnotes

1
https://www.fil.ion.ucl.ac.uk/spm/

2
http://www.rfmri.org/dpabi

## Abbreviations

svMCI: subcortical vascular mild cognitive impairment
fMRI: functional magnetic resonance imaging
DP: deficiency pattern
EP: excess pattern
PA: patient
CN: control
fALFF: fractional amplitude of low-frequency fluctuation
WMHs: white matter hyperintensities
rs-fMRI: resting-state functional magnetic resonance imaging
CM: Chinese medicine
SDSVD: syndrome differentiation score of vascular dementia
MoCA: Montreal Cognitive Assessment
TR: repetition time
TE: echo time
FA: flip angle
FOV: field of view
MNI: Montreal Neurological Institute
FWHM: full-width at half maximum
GS: global signals
WM: white matter
CSF: cerebrospinal fluid
FD: frame-wise displacement.
