# Supplementary material for: Lateralized brain activities in subcortical vascular mild cognitive impairment with differential Chinese medicine patterns: A resting-state functional magnetic resonance imaging study
Source: Front Neurosci. 2022 Aug 22;16:943929. doi: 10.3389/fnins.2022.943929 (PMC9441905; doi:10.3389/fnins.2022.943929)

**Supplementary Table 1 Brain regions that showed significant alterations in fALFF values (AlphaSim-corrected, p < 0.05)**

| **Brain Regions** | **R/L** | **Cluster Size** | **Peak MNI Coordinate** | | | **t value** |
| --- | --- | --- | --- | --- | --- | --- |
|  |  |  | ***x*** | ***y*** | ***z*** |  |
| **PA vs CN** | | | | | | |
| Cerebellum | R | 452 | 27 | -63 | -48 | 4.754 |
| Superior and Medial Frontal Gyrus | L | 225 | -27 | 21 | -15 | -3.558 |
| **DP vs CN** | | | | | | |
| Middle Frontal Gyrus | R | 224 | 36 | 9 | 60 | -3.641 |
| Cerebellum | R | 215 | 0 | -57 | -21 | 4.180 |
| **EP vs CN** | | | | | | |
| Orbit Frontal Gyrus | L | 178 | -3 | 45 | -12 | -4.804 |
| Cerebellum | L | 297 | -24 | -63 | -51 | 4.960 |
| **DP vs EP** | | | | | | |
| Middle Temporal Gyrus | R | 238 | 51 | 6 | -21 | 4.215 |
| Pons and Brainstem | / | 211 | -15 | -21 | -39 | 4.441 |
| Superior Temporal Gyrus | L | 438 | -57 | 6 | 0 | 4.801 |
| Anterior Cingulate and Paracingulate | R | 212 | 12 | 42 | 21 | -5.160 |

Abbreviations: fALFF: fractional amplitude of low-frequency fluctuation; PA: patient; DP: deficiency pattern; EP: excess pattern; CN: control; R: right; L: left; MNI: Montreal Neurological Institute; x, y, and z: coordinates of primary peak locations in MNI space.

**Supplementary Table 2 Correlation analysis**

| **Group** | **Brain Regions** |  | **Pearson Correlation** | |
| --- | --- | --- | --- | --- |
|  |  |  | ***r*** | ***p*** |
| DP vs CN | Right Middle Frontal Gyrus | DP＜CN | 0.544 | ＜0.001 |
|  | Right Cerebellum | DP﹥CN | -0.586 | ＜0.001 |
| EP vs CN | Left Orbit Frontal Gyrus | EP＜CN | -0.725 | ＜0.001 |
|  | Left Cerebellum | EP﹥CN | 0.649 | ＜0.001 |
| DP vs EP | Right Middle Temporal Gyrus | DP﹥EP | 0.047 | 0.789 |
|  | Pons and Brainstem | DP﹥EP | 0.108 | 0.538 |
|  | Left Superior Temporal Gyrus | DP﹥EP | 0.119 | 0.497 |
|  | Right Anterior Cingulate and Paracingulate | DP＜EP | 0.214 | 0.216 |

Abbreviations: DP: deficiency pattern; EP: excess pattern; CN: control

**Supplementary Fig 1 Brain regions that showed significantly altered fALFF values when comparing DPs with EPs** Abbreviations: fALFF: fractional amplitude of low-frequency fluctuation; DP: deficiency pattern; EP: excess pattern


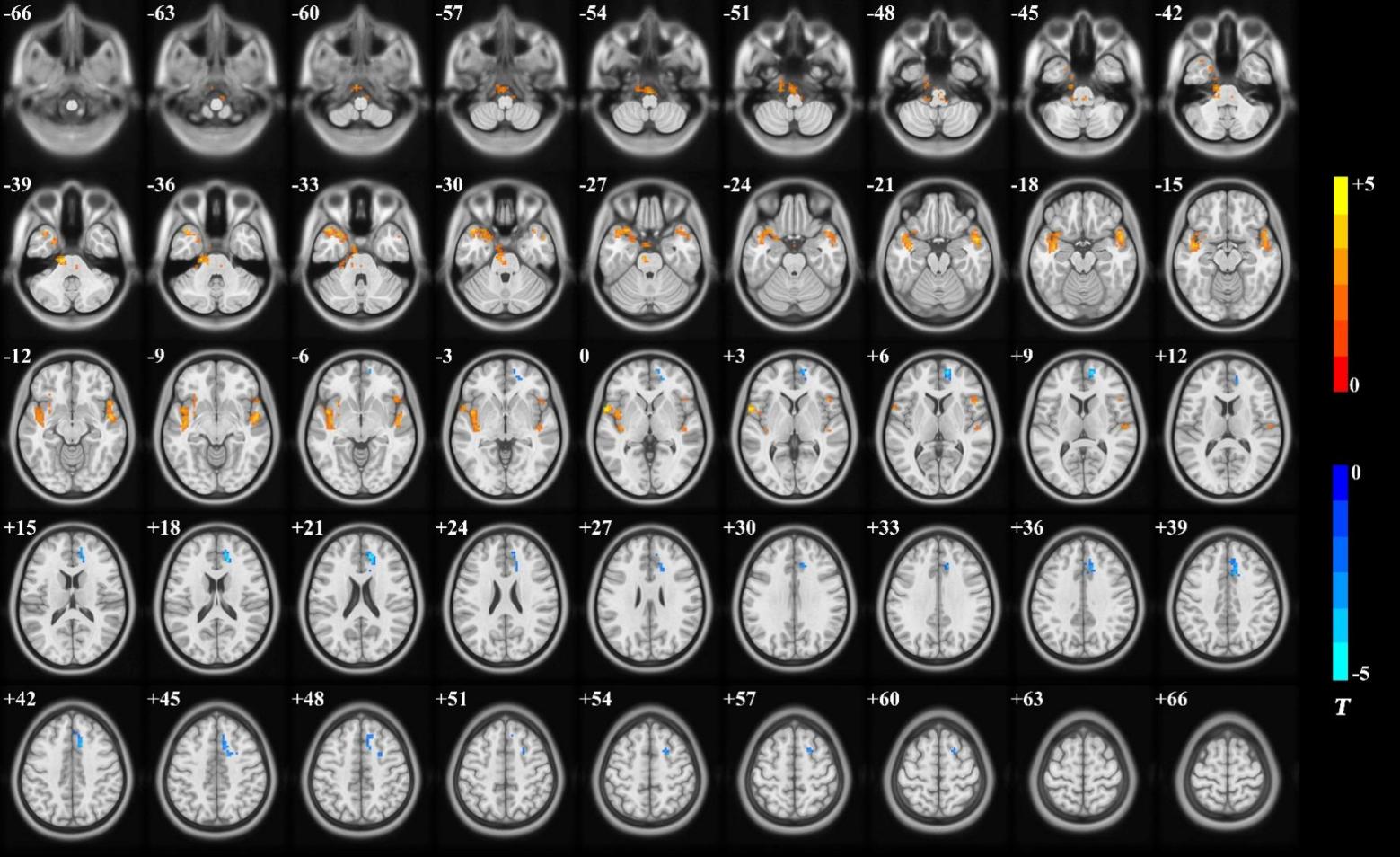

Supplement: Supplementary file 1 [file Data_Sheet_1.docx]
